# Histone deacetylases 1 and 2 cooperate in regulating BRCA1, CHK1, and RAD51 expression in acute myeloid leukemia cells

**DOI:** 10.18632/oncotarget.14062

**Published:** 2016-12-21

**Authors:** Jianyun Zhao, Chengzhi Xie, Holly Edwards, Guan Wang, Jeffrey W Taub, Yubin Ge

**Affiliations:** ^1^ National Engineering Laboratory for AIDS Vaccine and Key Laboratory for Molecular Enzymology and Engineering, The Ministry of Education, School of Life Sciences, Jilin University, Changchun, P. R. China; ^2^ Department of Oncology, Wayne State University School of Medicine, Detroit, MI, USA; ^3^ Molecular Therapeutics Program, Barbara Ann Karmanos Cancer Institute, Wayne State University School of Medicine, Detroit, MI, USA; ^4^ Department of Pediatrics, Wayne State University School of Medicine, Detroit, MI, USA; ^5^ Division of Pediatric Hematology/Oncology, Children's Hospital of Michigan, Detroit, MI, USA

**Keywords:** HDAC, BRCA1, CHK1, RAD51, acute myeloid leukemia

## Abstract

Resistance to chemotherapy and a high relapse rate highlight the importance of finding new therapeutic options for the treatment of acute myeloid leukemia (AML). Histone deacetylase (HDAC) inhibitors (HDACIs) are a promising class of drugs for the treatment of AML. HDACIs have limited single-agent clinical activities, but when combined with conventional or investigational drugs they have demonstrated favorable outcomes. Previous studies have shown that decreasing expression of important DNA damage repair proteins enhances standard chemotherapy drugs. In our recent studies, the pan-HDACI panobinostat has been shown to enhance conventional chemotherapy drugs cytarabine and daunorubicin in AML cells by decreasing the expression of BRCA1, CHK1, and RAD51. In this study, we utilized class- and isoform-specific HDACIs and shRNA knockdown of individual HDACs to determine which HDACs are responsible for decreased expression of BRCA1, CHK1, and RAD51 following pan-HDACI treatment in AML cells. We found that inhibition of both HDAC1 and HDAC2 was necessary to decrease the expression of BRCA1, CHK1, and RAD51, enhance cytarabine- or daunorubicin-induced DNA damage and apoptosis, and abrogate cytarabine- or daunorubicin-induced cell cycle checkpoint activation in AML cells. These findings may aid in the development of rationally designed drug combinations for the treatment of AML.

## INTRODUCTION

The standard treatment for most acute myeloid leukemia (AML) patients, consisting of cytarabine (ara-C) and an anthracycline [e.g., daunorubicin (DNR)], has been used for the past four decades. Though a high percentage of patients respond to induction therapy, a majority relapse [1]. AML is typically diagnosed in elderly individuals (median age 60–65 years); this population has higher rates of therapy-related relapse and decreased efficacy [2, 3]. Therefore, more effective therapies are urgently needed to improve treatment outcome of AML patients.

Histone deacetylase (HDAC) inhibitors (HDACIs) are a class of antileukemic agents of particular promise due to their effects on cell differentiation, cell cycle arrest, and apoptosis in human leukemic cells, but less so in normal cells [1, 4, 5]. However, the pleiotropic spectrum of pan-HDACIs makes it difficult to investigate the specific function of individual HDACs which contribute to the antileukemic effect. Although HDACIs have limited single-agent clinical activities [6–10], they have demonstrated promising results when combined with conventional and investigational drugs for the treatment of AML [11–13]. We previously demonstrated that panobinostat (a pan-HDACI) suppressed the expression of BRCA1, CHK1, and RAD51 which play critical roles in the DNA damage response (DDR), leading to induction of DNA DSBs and apoptosis, and abrogation of the activation of the cell cycle checkpoints induced by ara-C or DNR in AML cells [14]. However, it is still unknown which HDAC isoforms play a key role in regulating the expression of these proteins.

In this study, we demonstrate that together HDAC1 and HDAC2 activities are responsible for the decreased expression of BRCA1, CHK1, and RAD51 following class I- or pan-HDACI treatment in AML cells. By treating THP-1 and OCI-AML3 cell lines with class-, subclass-, and isoform-selective HDACIs, we found that simultaneous inhibition of HDAC1 and HDAC2 caused decreased expression of BRCA1, CHK1, and RAD51. Inhibition of HDAC1 and HDAC2 enhanced DNA DSBs and apoptosis induced by ara-C or DNR. Furthermore, it abrogated the activation of the S and G2 cell cycle checkpoints induced by ara-C or DNR in AML cell lines. These findings provide a better therapeutic strategy for the development of new HDACIs for the treatment of AML.

## RESULTS

### Class II HDACs are irrelevant with respect to the expression of *BRCA1, CHK1*, and *RAD51* in AML cells

**Figure 1:**
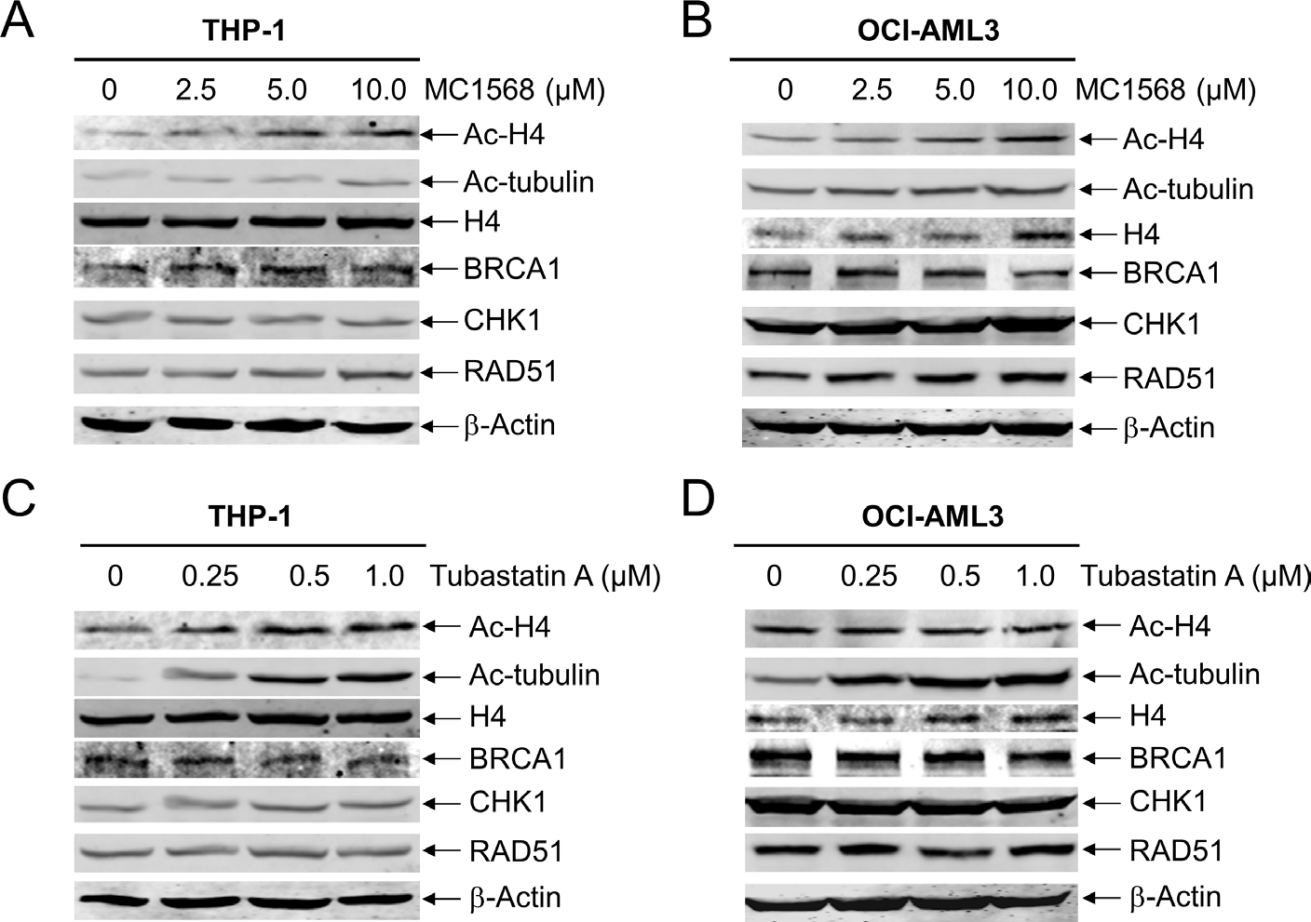
Inhibition of Class II HDACs has no impact on the expression of BRCA1, CHK1, and RAD51 in AML cells (**A** and **B**) THP-1 and OCI-AML3 cells were treated with MC1568 for 48 h, and then whole cell lysates were subjected to Western blotting and probed with the indicated antibodies. (**C** amd **D**) THP-1 and OCI-AML3 cells were treated with Tubastatin A for 48 h, and then whole cell lysates were subjected to Western blotting and probed with the indicated antibodies.

In our previous study, we demonstrated that the most potent pan-HDACI panobinostat induced apoptosis by suppressing the expression of DNA repair proteins BRCA1, CHK1, and RAD51 in AML cells [14]. Further, we found that inhibition of both HDACs 1 and 6 was critical for enhancing ara-C-induced apoptosis in pediatric AML cells [15]. To investigate which specific HDAC isoforms play critical roles in this process in AML cells, first we focused on Class II HDACs. We treated THP-1 and OCI-AML3 cell lines with variable concentrations of MC1568 (a Class IIa-selective HDACI) for 48 h and then subjected whole cell lysates to Western blotting. As shown in Figure 1A and 1B, MC1568 treatment resulted in increased expression of ac-H4, but had no obvious impact on the expression of ac-tubulin. Interestingly, the expression levels of BRCA1, CHK1, and RAD51 in the AML cell lines remained largely unchanged, demonstrating that class IIa HDACs are not involved in the expression of these DDR genes (Figure 1A and 1B). Similar results were obtained when THP-1 and OCI-AML3 cells were treated with variable concentrations of Tubastatin A (a HDAC6-selective inhibitor) for 48 h (Figure 1C and 1D). Taken together, these results demonstrate that Class II HDACs do not disrupt BRCA1, CHK1, and RAD51 expression in AML cells.

### Inhibiting HDACs 1, 2, and 3 decreases the transcript and protein levels of *BRCA1, CHK1*, and *RAD51* and induces apoptosis in AML cell lines

**Figure 2:**
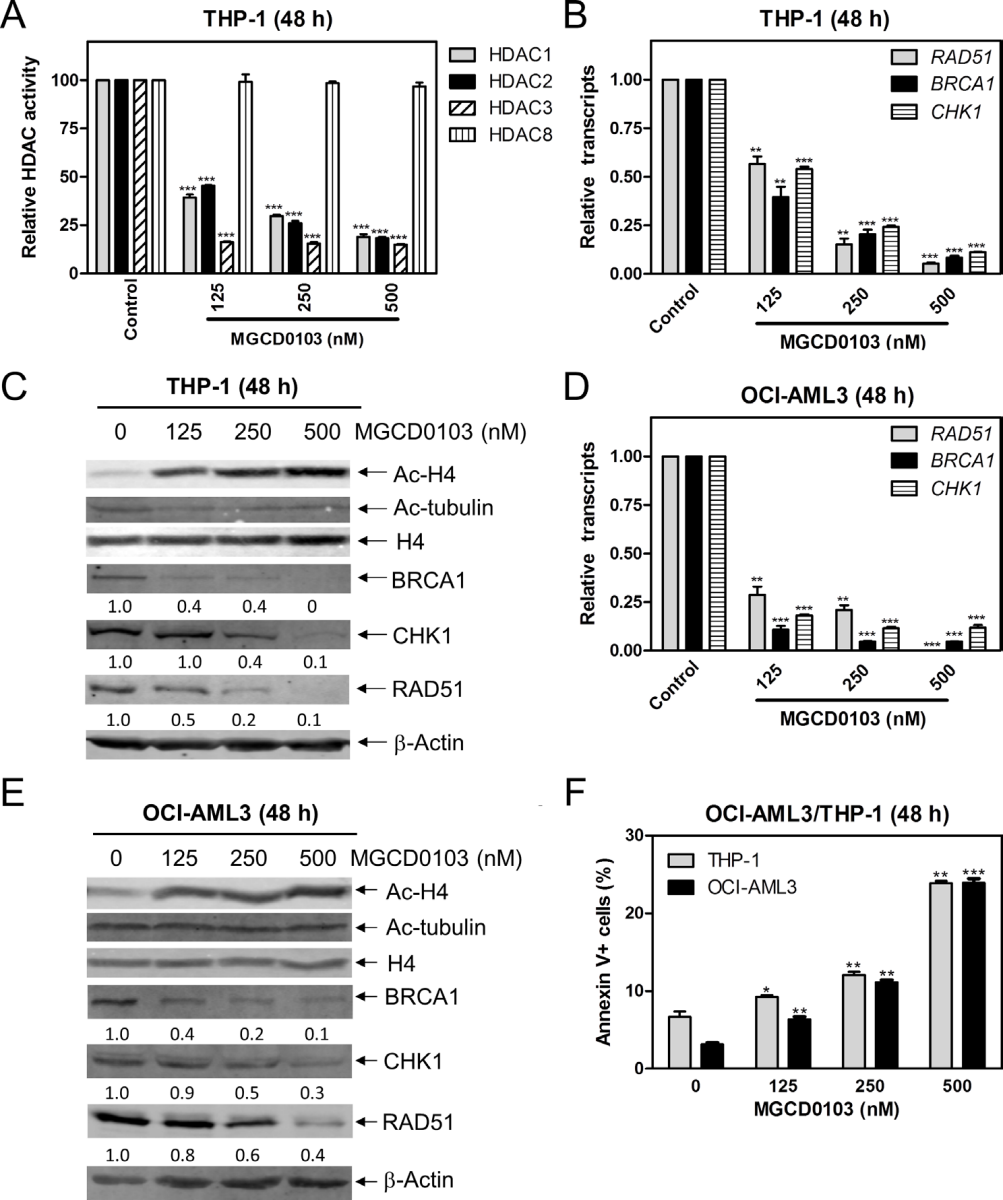
Inhibition of HDACs 1, 2, and 3 decreases the protein and transcript levels of BRCA1, CHK1, and RAD51, and induces apoptosis in AML cell lines (**A**) THP-1 cells were treated with variable concentrations of MGCD0103 for 48 h. Protein extracts were subjected to immunoprecipitation with antibodies against class I HDACs and then class I HDAC activities were measured, as described in the Materials and Methods. (**B**) THP-1 cells were treated with MGCD0103 for 48 h. Then total RNAs were isolated and gene transcript levels were determined by Real-time RT-PCR. Transcript levels were normalized to GAPDH and relative expression levels were calculated using the comparative Ct method. (**C**) THP-1 cells were treated with MGCD0103 for 48 h. Whole cell lysates were subjected to Western blotting and probed with the indicated antibodies. The fold changes for the densitometry measurements, normalized to β-actin and then compared to no drug treatment control, are indicated. (**D**) OCI-AML3 cells were treated with MGCD0103 for 48 h, then total RNAs were isolated from treated cells and gene transcript levels were determined by Real-time RT-PCR. Transcript levels were normalized to GAPDH and relative expression levels were calculated using the comparative Ct method. (**E**) OCI-AML3 cells were treated with MGCD0103 for 48 h. Whole cell lysates were subjected to Western blotting and probed with the indicated antibodies. (**F**) THP-1 and OCI-AML3 cells were treated with MGCD0103 for 48 h and then subjected to Annexin V-FITC/PI staining and flow cytometry analysis. *indicates *p* < 0.05, **indicates *p* < 0.01, and ***indicates *p* < 0.001 (panels A, B, D & F).

To determine if Class I HDACs affect the transcript and protein levels of *BRCA1*, *CHK1*, and *RAD51* genes, we treated THP-1 cells with variable concentrations of MGCD0103 (a class I HDACI) for 48 h and then measured the enzymatic activities of HDACs 1, 2, 3, and 8 following immunoprecipitation. MGCD0103 caused significant inhibition of HDACs 1, 2, and 3 activities, but did not affect HDAC8 activity (Figure 2A). Then we measured *BRCA1*, *CHK1*, and *RAD51* transcript levels by real-time RT-PCR and protein levels by Western blotting in the cell lines post MGCD0103 treatment. There was a concentration-dependent decrease of *BRCA1*, *CHK1*, and *RAD51* transcript and protein levels in THP-1 cells (Figure 2B and 2C). Meanwhile, MGCD0103 caused concentration-dependent increase of acetylated-histone H4, while having no effect on acetylation of alpha-tubulin and total histone H4 levels (Figure 2C). Similar results were also obtained in OCI-AML3 cells (Figure 2D–2F). Interestingly, downregulation of these DDR genes by MGCD0103 treatment was accompanied by concentration-dependent induction of apoptosis in both cell lines (Figure 2F). Together, these results demonstrate that simultaneous inhibition of HDACs 1, 2, and 3 by MGCD0103 suppresses the transcript and protein expression levels of *BRCA1*, *CHK1*, and *RAD51* in AML cell lines.

### Inhibiting HDACs 1, 2, and 3 enhances the antileukemic activities of ara-C and DNR against AML cells

**Figure 3:**
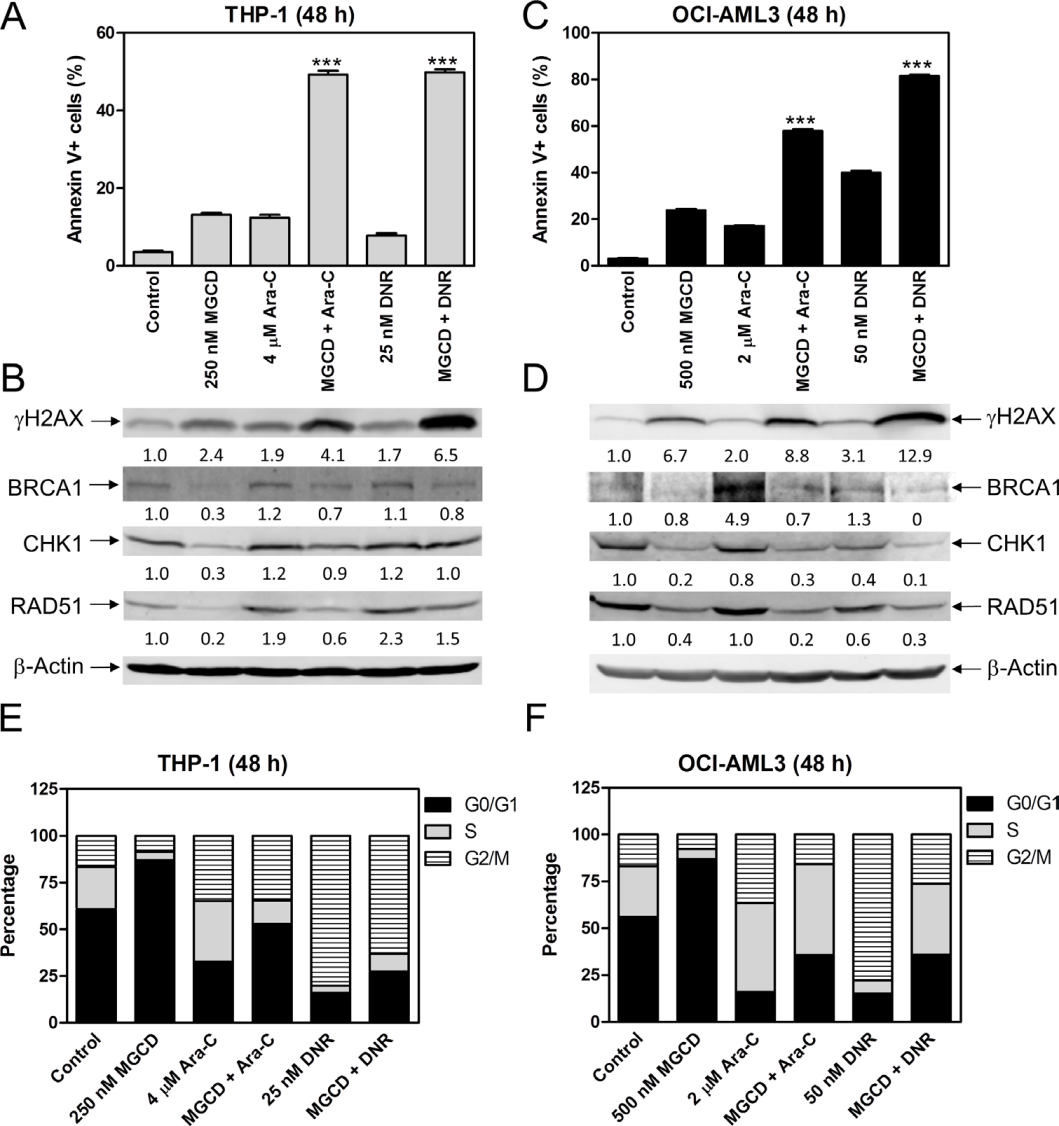
MGCD0103 cooperates with ara-C or DNR in inducing apoptosis and abrogates S and/or G2/M cell cycle checkpoint activation induced by ara-C or DNR in AML cells (**A** and **C**) THP-1 and OCI-AML3 cells were treated with MGCD0103 and ara-C or DNR, alone or in combination, for 48 h and then subjected to Annexin V-FITC/PI staining and flow cytometry analyses. ***indicates *p* < 0.001. (**B** and **D**) Whole cell lysates were subjected to Western blotting and probed with the indicated antibodies. The fold changes for the densitometry measurements, normalized to β-actin and then compared to no drug treatment control, are indicated. (**E** and **F**) THP-1 and OCI-AML3 cells were treated for 48 h with MGCD0103 and ara-C or DNR, alone or in combination, then fixed with ethanol and stained with PI for cell cycle analysis.

To determine if inhibiting HDAC1, HDAC2, and HDAC3 enhances the antileukemic activity of ara-C or DNR, we treated THP-1 and OCI-AML3 cells with MGCD0103 and ara-C or DNR, alone or combined, for 48 h and then subjected the cells to Annexin V/propidium iodide (PI) staining, and flow cytometry. Consistent with panobinostat, MGCD0103 enhanced ara-C- and DNR-induced apoptosis in THP-1 and OCI-AML3 cells (Figure 3A and 3C), which was accompanied by increased DNA DSBs, as reflected by the induction of γH2AX (an established biomarker for DNA double-strand breaks [16], Figure 3B and 3D). In the combined treatment, we detected decreased expression of BRCA1, CHK1, and RAD51 compared to ara-C or DNR alone in both cell lines (Figure 3B and 3D). MGCD0103 treatment alone resulted in increased G0/G1 phase cells, suggesting G0/G1 arrest and ara-C treatment resulted in increased S and G2/M phase cells, suggesting S and G2/M arrest in both cell lines. In THP-1 cells, ara-C-induced increase of S phase cells was reduced, while in OCI-AML3 cells ara-C-induced increase of G2/M phase cells was decreased by the addition of MGCD0103 (Figure 3E and 3F). DNR treatment resulted in increased G2/M phase cells, which was reduced by co-administration of MGCD0103 in both cell lines (Figure 3E and 3F). These results suggest that inhibition of HDACs 1, 2, and 3 enhances the antileukemic activity of ara-C and DNR through suppressing the expression of BRCA1, CHK1, and RAD51 in AML cells.

### HDACs 1 and 2 cooperate in regulating the expression of *BRCA1, CHK1*, and *RAD51* in AML cells

**Figure 4:**
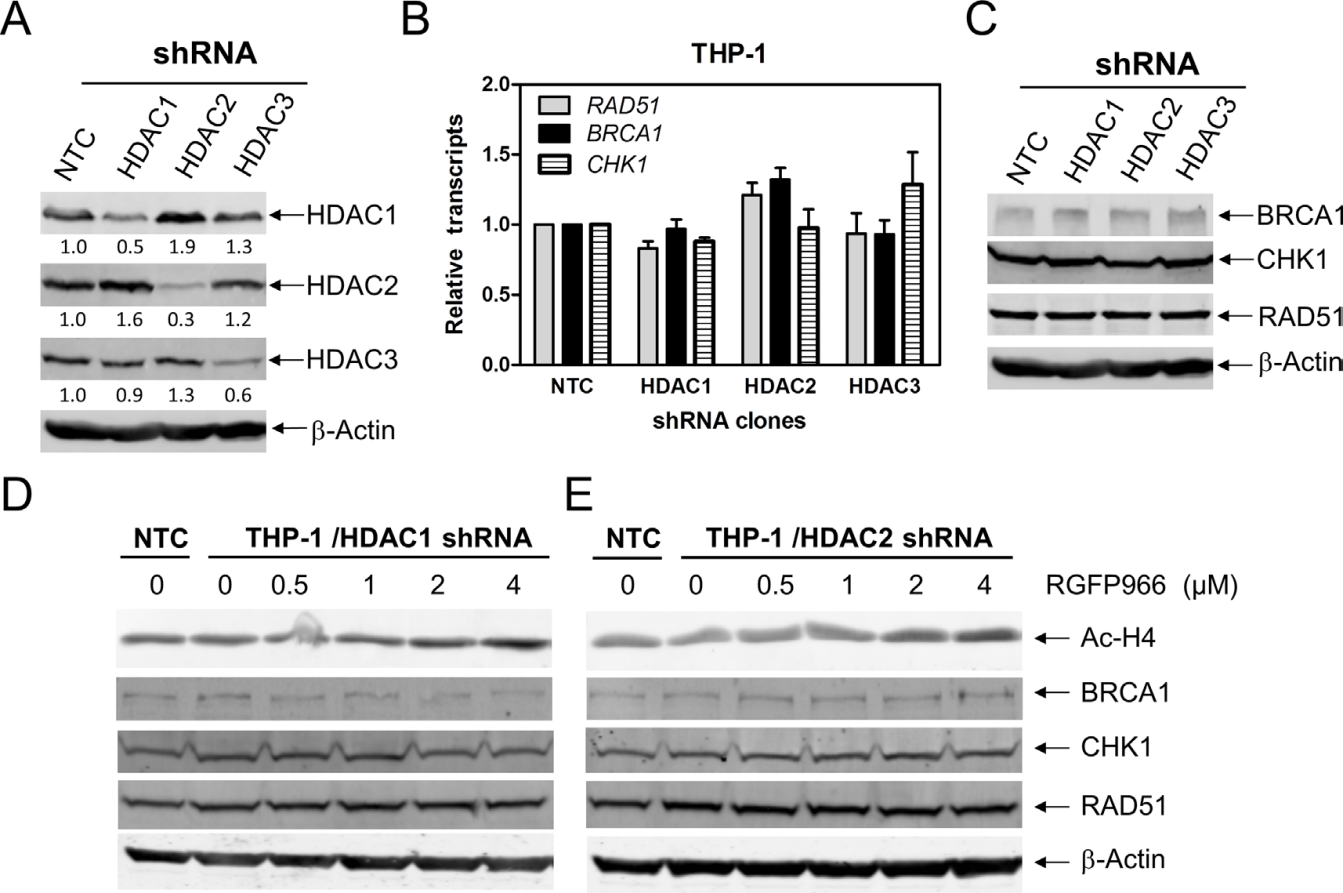
HDACs 1 and 2 cooperate in regulating BRCA1, CHK1, and RAD51 expression in AML cells (**A** and **C**) THP-1 cells were infected with HDAC1 (THP-1/HDAC1), HDAC2 (THP-1/HDAC2), HDAC3 (THP-1/HDAC3), or NTC control (THP-1/NTC) shRNA lentivirus overnight, then washed and incubated for 48 h prior to adding puromycin to the culture medium. Whole cell lysates were subjected to Western blotting and probed with the indicated antibodies. The fold changes for the densitometry measurements, normalized to β-actin and then compared to no drug treatment control, are indicated. These Western blots were previously published [15]. (**B**) Total RNAs were isolated from the transfected cells and gene transcript levels were determined by Real-time RT-PCR. Transcript levels were normalized to GAPDH and relative expression levels were calculated using the comparative Ct method. (**D** and **E**) THP-1/HDAC1 and THP-1/HDAC2 cells were treated with RGFP966 for 48 h. Whole cell lysates were subjected to Western blotting and probed with the indicated antibodies.

**Figure 5:**
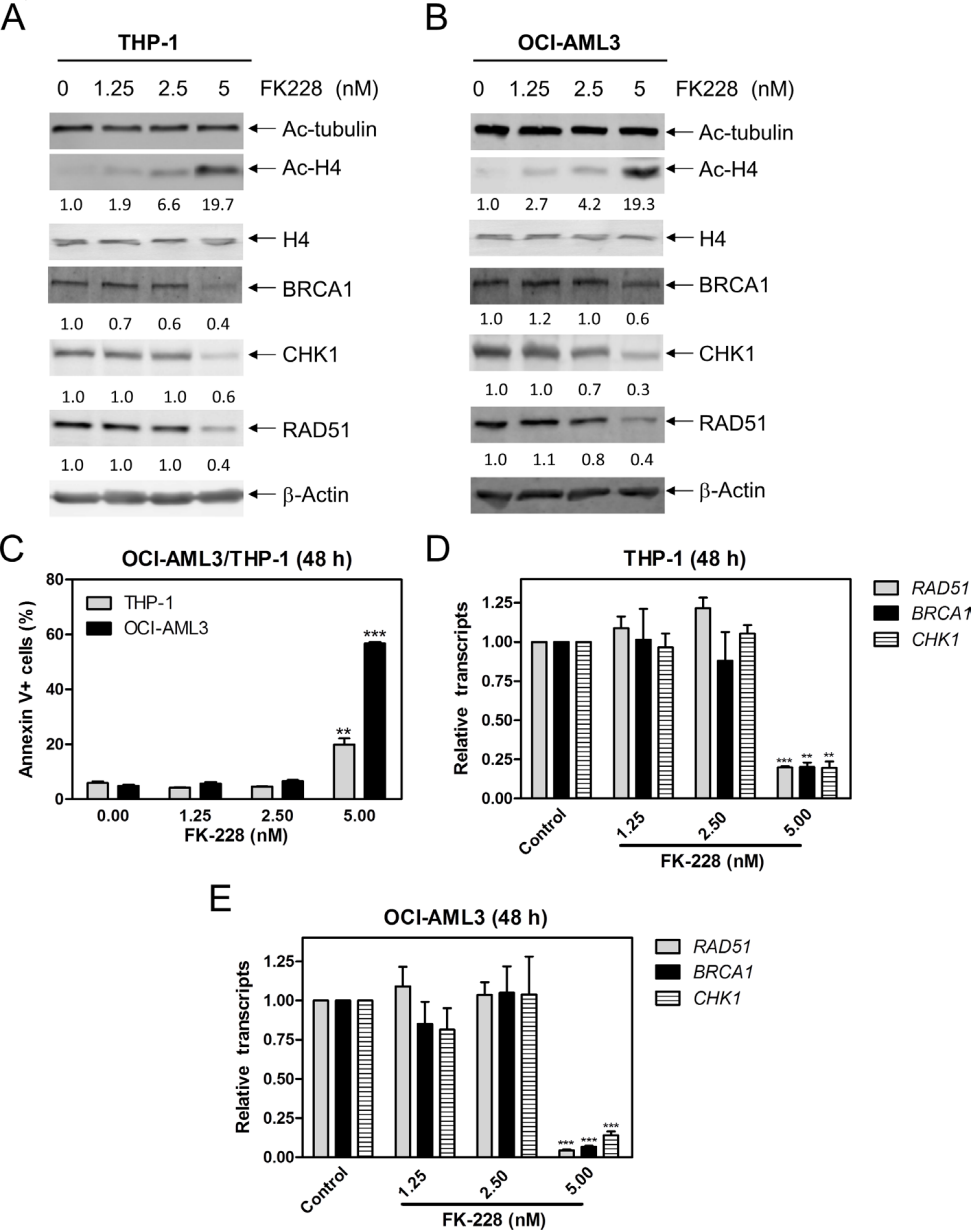
FK228 decreases expression of BRCA1, CHK1, and RAD51 by inhibiting HDAC1 and HDAC2 in AML cells (**A** and **B**) THP-1 and OCI-AML3 cells were treated with FK228 for 48 h and whole cell lysates were subjected to Western blotting and probed with the indicated antibodies. The fold changes for the densitometry measurements, normalized to β-actin and then compared to no drug treatment control, are indicated. (**C**) THP-1 and OCI-AML3 cells were treated with FK228 for 48 h and then subjected to Annexin V-FITC/PI staining and flow cytometry analyses. **indicates *p* < 0.01 and ***indicates *p* < 0.001. (**D** and **E**) THP-1 and OCI-AML3 cells were treated with FK228 for 48 h. Total RNAs were isolated from treated cells and gene transcript levels were determined by Real-time RT-PCR. Transcript levels were normalized to GAPDH and relative expression levels were calculated using the comparative Ct method. **indicates *p* < 0.01 and ***indicates *p* < 0.001.

To further investigate which HDAC isoforms, among HDAC1, HDAC2, and HDAC3, play important roles in regulating expression of *BRCA1*, *CHK1*, and *RAD51* in AML cells, we individually knocked down *HDAC1*, *HDAC2*, and *HDAC3* in THP-1 cells (Figure 4A, designated THP-1/HDAC1, THP-1/HDAC2, and THP-1/HDAC3, respectively; Western blot verification of knockdown was previously published [15]). Then we determined BRCA1, CHK1, and RAD51 transcript and protein levels in these cells by real-time RT-PCR and Western blotting, respectively. As show in Figure 4B and 4C, *HDAC1*, *HDAC2* or *HDAC3* knockdown did not affect *BRCA1*, *CHK1*, and *RAD51* transcript and protein levels in THP-1 AML cells. Next we treated THP-1/HDAC1 cells and THP-1/HDAC2 cells with variable concentrations of RGFP966, an HDAC3-selective inhibitor, for 48 h, and then subjected the cells to Western blotting. As shown in Figure 4D and 4E, RGFP966 treatment had no impact on the expression of BRCA1, CHK1, and RAD51 compared to no drug treated control or the no drug treated NTC cells. These results suggest that simultaneous inhibition of HDAC1 and HDAC3 or HDAC2 and HDAC3 is not sufficient to reduce BRCA1, CHK1, and RAD51 expression. Then we treated THP-1 and OCI-AML3 cells with variable concentrations of FK228 (an HDAC1 and HDAC2 inhibitor) for 48 h. Suppression of *BRCA1*, *CHK1*, and *RAD51* transcript and protein expression was detected after cells were treated with 5 nM FK228, which was accompanied by apoptosis (Figure 5). These results revealed that HDAC1 and HDAC2 cooperate in regulating *BRCA1*, *CHK1*, and *RAD51* transcript and protein expression in AML cells.

### Simultaneous inhibition of *HDAC1* and *HDAC2* enhances the antileukemic activities of ara-C and DNR against AML cells

**Figure 6:**
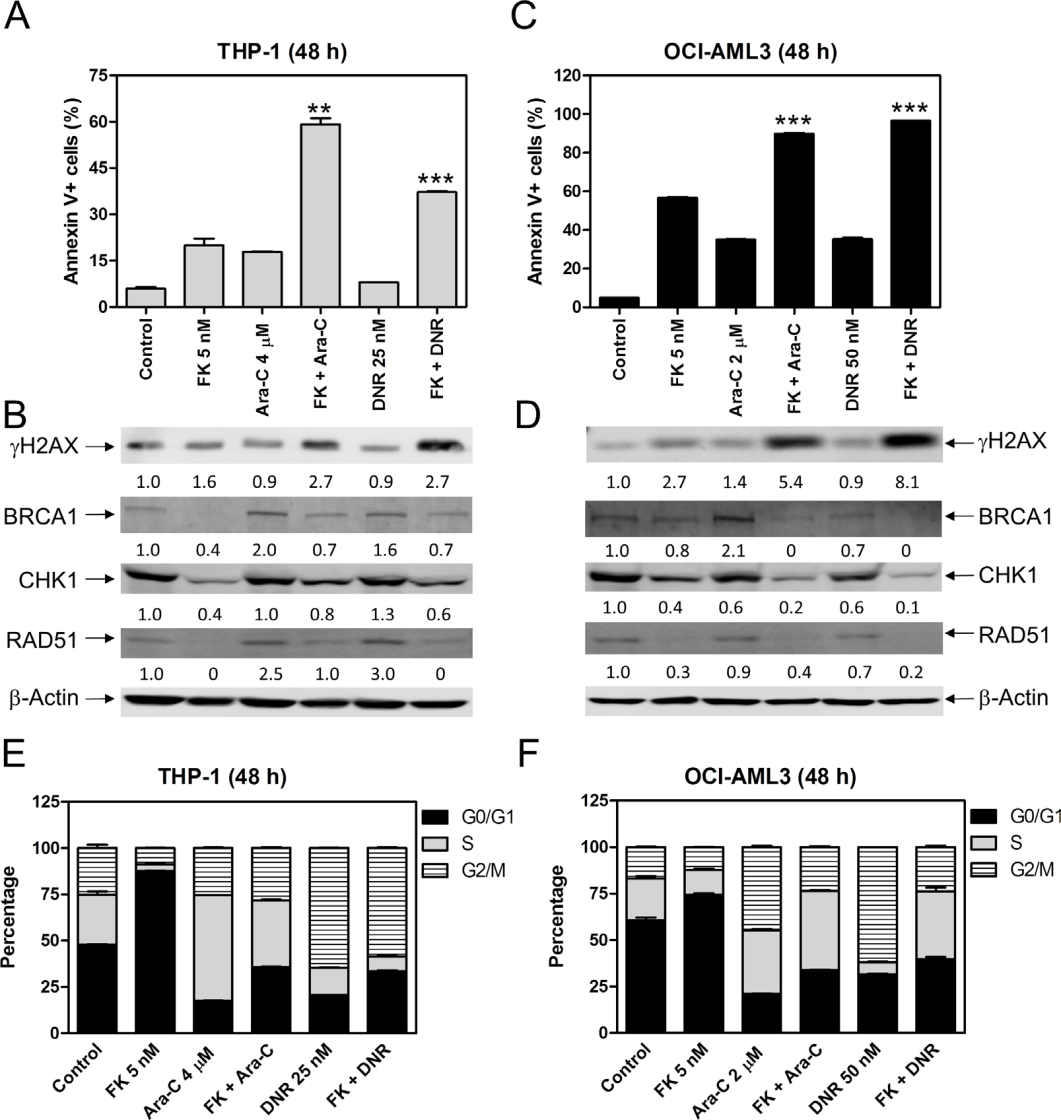
FK228 cooperates with ara-C or DNR in inducing apoptosis and abrogates S and/or G2/M cell cycle checkpoint activation induced by ara-C or DNR in THP-1 and OCI-AML3 AML cells (**A**) THP-1 cells were treated with FK228 and ara-C or DNR, alone or in combination, for 48 h and then subjected to Annexin V-FITC/PI staining and flow cytometry analyses. **indicates *p* < 0.01 and ***indicates *p* < 0.001. (**B**) Whole cell lysates were subjected to Western blotting and probed with the indicated antibodies. The fold changes for the densitometry measurements, normalized to β-actin and then compared to no drug treatment control, are indicated. (**C**) OCI-AML3 cells were treated with FK228 and ara-C or DNR, alone or in combination, for 48 h and then subjected to Annexin V-FITC/PI staining and flow cytometry analyses. ***indicates *p* < 0.001. (**D**) Whole cell lysates were subjected to Western blotting and probed with the indicated antibodies. The fold changes for the densitometry measurements, normalized to β-actin and then compared to no drug treatment control, are indicated. (**E** and **F**) THP-1 and OCI-AML3 cells were treated with FK228 and ara-C or DNR, alone or in combination for 48 h and then fixed with ethanol and stained with PI for cell cycle analysis.

To determine the effects of HDAC1 and HDAC2 on the antileukemic activity of ara-C and DNR, we treated THP-1 and OCI-AML3 cells with FK228 and ara-C or DNR, alone or in combination, for 48 h. The cells were then subjected to flow cytometry analyses for apoptosis and cell cycle progression. The results revealed that FK228 can enhance ara-C- and DNR-induced apoptosis in THP-1 and OCI-AML3 cells (Figure 6A and 6C), accompanied by reduced expression of BRCA1, CHK1, and RAD51 compared to ara-C or DNR alone and increased DNA DSBs, as reflected by the induction of γH2AX (Figure 6B and 6D). FK228 treatment alone resulted in increased G0/G1 phase cells in THP-1 and OCI-AML3 cells. FK228 partially abrogated ara-C-induced increase of S phase cells in THP-1 cells and decreased ara-C-induced increase of G2/M phase cells in OCI-AML3 cells. DNR-induced increase of G2/M cells was reduced by co-administration with FK228 in both cell lines (Figure 6E and 6F). These results are similar to those following MGCD0103 treatment, suggesting that inhibiting HDAC1 and HDAC2 has the same effect of decreasing the expression of BRCA1, CHK1, and RAD51, as inhibiting HDAC1, HDAC2 and HDAC3 together.

## DISSCUSSION

In our previous study, we demonstrated that panobinostat (pan-HDACI) suppresses the expression of three critical DDR proteins, BRCA1, CHK1, and RAD51, leading to enhancement of DNA DSBs, abrogation of cell cycle checkpoints, and enhanced induction of apoptosis by ara-C or DNR in AML cells [14]. We also found that inhibition of both HDACs 1 and 6 was critical in enhancing ara-C-induced apoptosis in pediatric AML cells [15]. However, which HDAC isoforms participate in regulating the expression of BRCA1, CHK1, and RAD51 in AML cells remains unknown. Solving this problem could help us better understand the specific function of individual HDACs, which is essential for the development of new therapies, as well as for rationally designing combination therapies for the treatment of AML.

Treatment with a Class II HDACI, MC1568 or Tubastatin A, had no effect on BRCA1, CHK1, or RAD51 protein levels, demonstrating that Class II HDACs do not have an impact on the expression of these proteins. The Class I HDACI MGCD0103, which inhibits HDACs 1, 2, and 3, caused concentration-dependent decrease of both protein and transcript levels for *BRCA1*, *CHK1*, and *RAD51* in THP-1 and OCI-AML3 cells. MGCD0103 enhanced DNA DSBs and apoptosis induced by ara-C or DNR, and it also abrogated S and/or G2/M checkpoint activation induced by ara-C or DNR in these cells (Figure 3). These results are consistent with our previous findings using panobinostat [14], suggesting that HDAC1, HDAC2, and HDAC3 participate in the regulation of BRCA1, CHK1, and RAD51 expression and play important roles in the antileukemic activities of ara-C or DNR in AML cells.

While individual knockdown of *HDAC1*, *HDAC2* or *HDAC3* did not alter *BRCA1*, *CHK1*, and *RAD51* expression, combined inhibition of HDAC1 and HDAC2 decreased their expression. Our results were consistent with Miller et al. who demonstrated that HDAC1 and HDAC2 promote double-strand break repair [17], though the mechanism was not determined. It has been reported that inhibition of HDAC2 causes downregulation of RAD51 in melanoma cells [18]. Thurn and colleagues have reported that inhibition or siRNA knockdown of both *HDAC1* and *HDAC2* not only resulted in decreased BRCA1 transcript levels, but also ATM transcript and protein levels as well [19]. Although we did not investigate ATM levels, HDAC1 and HDAC2 inhibition caused decreased transcript and protein levels of *BRCA1*, *CHK1*, and *RAD51* in AML cells.

In summary, our study demonstrates that simultaneous inhibition of both HDAC1 and HDAC2 decreases the expression of *BRCA1*, *CHK1*, and *RAD51*, induces DNA DSBs and apoptosis, and abrogates cell cycle checkpoint activation induced by ara-C or DNR in AML cells. pan-HDACIs have been shown to decrease *BRCA1*, *CHK1*, and *RAD51* expression through transcriptional regulation, of which E2F1 plays a critical role [14, 20], and post-translational mechanisms [21]. In addition, in AML cells it has been shown that inhibition of HDAC1 and HDAC2 causes upregulation of miR-182 which directly targets RAD51, resulting in decrease of RAD51 expression [22]. These studies indicate that HDAC1 and HDAC2 regulate the expression of *BRCA1*, *CHK1*, and *RAD51* through both transcriptional and post-translational mechanisms. Our data support combining a class I HDACI with DNA damaging agents for the treatment of AML and provide guidance for the further development of HDAC selective inhibitors.

## MATERIALS AND METHODS

### Drugs

MGCD0103, MC1568, Tubastatin A, RGFP966, and FK228 (also called Romidepsin or depsipeptide) were purchased from Selleck Chemicals (Houston, TX). Daunorubicin (DNR) and cytarabine (Ara-C) were purchased from Sigma-Aldrich (St. Louis, MO).

### Cell culture

The THP-1 cell line was purchased from the American Type Culture Collection (Manassas, VA). The OCI-AML3 cell line was purchased from the German Collection of Microorganisms and Cell Cultures (DSMZ, Braunschweig, Germany). The THP-1 cell line was cultured in RPMI 1640 and the OCI-AML3 cell line was cultured in alpha-MEM with 10–15% fetal bovine serum (Life Technologies, Grand Island, NY), 2 mM L-glutamine, 100 U/ml penicillin and 100 μg/ml streptomycin. All cells were cultured in a 37°C humidified atmosphere containing 5% CO2/95% air.

### Enzymatic assays of class I HDACs following immunoprecipitation (IP)

THP-1 cells were treated with variable concentrations of MGCD0103 for 48 h and lysed in Cell Lysis Buffer [20 mmol/L Tris-HCl (pH 8), 0.15 mol/L NaCl, 10% glycerol, and 0.5% NP40] on ice for 2 hours. After centrifugation (12,000 × g for 15 minutes), 500 μg of the supernatant fraction (cell lysate) was incubated with 2 μg rabbit IgG, anti-HDAC1, anti-HDAC3 (Bethyl Labs, Montgomery, TX), anti-HDAC2 (CycLex, Nagano, Japan) or 1000 μg of supernatant fraction was incubated with 2 μg anti-HDAC8 (Santa Cruz Biotechnology, California) overnight at 4°C, followed by incubation with 30 μl of Protein A/G Dynabeads (Life Technologies) for 3 hours at 4°C. The beads were washed three times with ice cold PBS and resuspended in HDAC Assay Buffer [40 mL; 20 mmol/L Tris-HCl (pH 8), 125 mmol/L NaCl, and 1% glycerol] and then HDAC enzymatic activities were measured using the CycLexH HDACs Deacetylase Fluorometric Assay kit (CycLex), or heated at 95°C for 5 min in 30 μl loading buffer for Western blotting.

### Quantification of gene expression by real-time RT-PCR

Total RNA was extracted using TRIzol (Life Technologies), cDNAs were prepared from 2 μg total RNA using random hexamer primers and a RT-PCR kit (Life Technologies), and purified using the QIAquick PCR Purification Kit (Qiagen, Valencia, CA), as previously described [23, 24]. Transcripts for *BRCA1, CHK1*, and *RAD51* were quantitated using TaqMan probes (Life Technologies) and a LightCycler^®^ 480 real-time PCR machine (Roche Diagnostics, Indianapolis, IN), based on the manufacturer's instructions. Real-time PCR results are expressed as mean values from 3 independent experiments using the same cDNA preparations and were normalized to GAPDH. Fold changes were calculated using the comparative Ct method [25].

### Western blot analysis

Cells were lysed in the presence of protease and phosphatase inhibitors (Roche Diagnostics). Whole cell lysates were subjected to SDS-polyacrylamide gel electrophoresis, electrophoretically transferred onto polyvinylidene difluoride (PVDF) membranes (Thermo Fisher Inc., Rockford, IL), and immunoblotted with anti-acetyl-histone 4 (ac-H4), -H4, -acetyl-tubulin (ac-tubulin, Upstate Biotechnology, Lake Placid, NY), -γH2AX, -HDAC1, -HDAC2, -HDAC3 (Cell Signaling Technology), -BRCA1, -RAD51, -CHK1 (Santa Cruz Biotechnology) or -β-actin (Sigma, St Louis, MO) antibody, as previously described [26, 27]. Immunoreactive proteins were visualized using the Odyssey Infrared Imaging System (Li-Cor, Lincoln, NE, USA).

### Apoptosis

AML cells were treated with the indicated drugs for 48 h and then subjected to flow cytometry analysis to determine drug-induced apoptosis using an Annexin V-fluorescein isothiocyanate (FITC)/PI apoptosis Kit (Beckman Coulter; Brea, CA, USA), as previously described [24, 28]. Experiments were performed 3 independent times in triplicates. Results from one representative experiment are shown.

### Cell cycle progression

Cells were treated with the indicated drugs for 48 h. The cells were harvested and fixed with ice-cold 80% (v/v) ethanol for 24 h. The cells were pelleted, washed with PBS, and resuspended in PBS containing 50 μg/mL PI, 0.1% Triton X-100 (v/v), and 1 μg/mL DNase-free RNase. DNA content was determined by flow cytometry analysis using a FACS Calibur flow cytometer (Becton Dickinson), as previously described [14]. Cell cycle analysis was performed using ModFit LT 3.0 (Becton Dickinson).

### shRNA knockdown of *HDAC1, HDAC2*, and *HDAC3* in THP-1 cells

HDAC1, HDAC2, HDAC3, and non-target control (NTC) shRNA lentiviral particles were purchased from Sigma-Aldrich and used to infect THP-1 cells. THP-1 cells were transduced overnight and then cultured for an additional 48 h prior to selection with puromycin. A pool of infected cells was expanded and tested for HDAC1, HDAC2, and HDAC3 expression by Western blotting. A pool of cells from the NTC shRNA lentiviral transduction was used as the negative control.

### Statistical analysis

Differences in cell apoptosis and *BRCA1*, *CHK1*, and *RAD51* transcript levels between treated (individually or combined) and untreated cells were compared using the paired t-test. Statistical analyses were performed with GraphPad Prism 5.0. Error bars represent ± SEM. The level of significance was set at *p* < 0.05.
